# A qualitative exploration of “empathic labor” in Chinese hospice nurses

**DOI:** 10.1186/s12904-022-00911-w

**Published:** 2022-02-16

**Authors:** Ya-Ling Wang, Zi-Wei Yang, Yue-Zhong Tang, Hui-Ling Li, Lan-Shu Zhou

**Affiliations:** 1 School of Nursing, Soochow University, No.1, Shizi Street, Suzhou, 215000 China; 2 KangJian Community Health Service Center, 88 Jiangan Road, Shanghai, 200433 China; 3 School of Nursing, Naval Military Medical University, 800 Xiangyin Road, Shanghai, 200433 China

**Keywords:** Hospice care, Nurses, Empathy, Emotional labor, China

## Abstract

**Background:**

Hospice nurses may devote more emotional labor during the empathy process with patients, and this empathy can be used as a form of psychological behavior of emotional labor in the hospice care model. The aim of this study was to analyze hospice nurses’ empathy characteristics in the context of emotional labor theory, and explore the impact of empathy on patient care.

**Methods:**

We conducted semi-participant observations from three hospitals and multicenter in-depth interviews with *n* = 26 hospice nurses from eight cities. Interviews were transcribed, and directed content analysis was applied.

**Results:**

Two categories with four sub-categories were extracted from the data analysis. Category 1 described the “empathic labor” process which covers cognitive empathy (including empathic imagination, empathic consideration, and empathic perception) and affective empathy (including natural empathy, surface empathy, and deep empathy). The second category concerns the outcome of nurses’ “empathic labor” which incorporates both positive and negative effects.

**Conclusions:**

The findings indicated that hospice nurses’ empathy process should be understood as emotional labor. Nursing managers should pay more attention to raising the ability of deep empathy with hospice nurses, and explore more sufficient active empowerment strategies to alleviate the negative impact of empathy on nurses and to strengthen nurses' deep empathy with terminal ill patients.

## Background

Palliative care as defined by the World Health Organization (WHO) is an approach to improve the quality of life of patients with life-threatening illnesses and their families. The WHO points out that an estimated 40 million people are in need of palliative care; however, only approximately 14% of people who need palliative care currently receive palliative care worldwide [1]. At present, China is facing the dilemma of increasingly aging demographic change [2]. Thus, the need for palliative care is growing commensurately, and enabling nationwide access to palliative care is of special importance, as China aims to reach health care standards comparable to those in developed countries by 2030 [3]. To achieve this goal, China launched a pilot project of hospice care in some cities in 2017. Although the Chinese government has increased its support for hospice care in recent years, however, the development of hospice care in China is insufficient, which is mainly reflected in the lack of education around hospice care, inadequate composition of hospice care teams, unbalanced geographical distribution, and so on [4, 5].

As the core working members of the multidisciplinary collaborative team model of hospice care, nurses play an important role in the health care of end-of-life stage patients and their families. Empathy is considered to be vital to professional helping and caring relationships in hospice care, which requires nurses to have the ability to think from the perspective of patients [6]. According to empathy theory, nurses can make patients feel valued and recognized through affective empathy or cognitive empathy, which can help to promote trust and strengthen the caring relationship between nurses and patients [7]. However, previous studies have shown that hospice nurses have more opportunities to face the pain and death of patients during the empathy process [6], need to devote more emotional care to patients. It was reported that 78.3% hospice nurses or other medical staff in China believed that caring for a dying person can cause them to feel not only physically and emotionally exhausted but also inevitably have a strong sense of emotional trauma such as loss, anxiety, fear, and depression [4], which in turn made them empathic fatigue or reluctant to participate in hospice care [8, 9], even directly affects the quality of hospice service and threatens the stability of the hospice nursing team.

There are many different concepts of empathy that exist in the literature. These concepts come from various fields and are often radically discontinuous with one another [10]. As mentioned in some studies, nurses’ empathy is considered to be a form of psychological behavior of emotional labor in the hospice care [11, 12]. Emotional labor is described as a requirement of employment in many professions which require individuals to display certain emotions as part of their job performance, such as welcoming, cheerful, patient, sympathetic or always convey a sense of security and safety [13, 14]. There are four major techniques in the model of emotional labor, including natural action, surface action, deep action, and deliberative dissonance action [15]. To a nurse, the emotional technique is as important as clinical technique [16]. For oncology nurses, their emotional labor refers to a special bond help to establish a relationship with patients through empathy [17], and previous studies pointed out that empathy should be transformed into the form of emotional labor, and on this basis, corresponding training, practice, and research should be carried out [11, 12].

However, prior researches [18, 19] always focused on emphasizing the importance of strengthening terminal care performance of clinical nurses by improving their empathy ability or analyzed the relationship between burnout and empathy among hospice nurses. Few researches have studied the empathy of nurses as the form of emotional labor and explored nurses’ experiences of empathy in daily clinical work, especially for those in hospice nurses. To fill this gap, we conducted a qualitative descriptive study to improve the comprehension of empathy under the theory of emotional labor from the viewpoint of hospice nurses experiencing it. We also aimed to analyze hospice nurses’ empathy characteristics in the context of emotional labor theory, and explore the impact of empathy on patient care to provide strategies for improving nurses’ empathy ability.

## Methods

### Study design and setting

This was a descriptive qualitative study using semi-participant observations and multicenter in-depth interviews. Different from the participant observation, semi-participant observation allows the researcher to adopt “participant as observer” as his/her observation role, which means the researcher is known as an observer to participants, he/she establishes a relationship and participates in some activities with participants [20, 21]. Before and during the multicenter in-depth interviews, we explored the empathy behavior of hospice nurses in clinical work with field research in August 2020, October 2020, and February 2021. Interviews were conducted either in person or via video chat software to further elaborate and explore the perceptions and experiences of the “empathic labor” with the hospice nurses between December 2020 to February 2021. Ethical approval was granted by the Medical Ethics Committee of The First Affiliated Hospital of Soochow University, China.

### Field observation

We selected three hospice units in Shanghai, Shenzhen, and Lianyungang as the field sites by using purposive sampling. The researcher entered those three field sites as a "hospice trainee nurse" for one week of observation after obtaining informed consent from the heads of the units. Field notes were obtained in three observation phases, from descriptive observation to focused observation to selective observation [22], which can help to support the data analysis and develop the themes and subthemes. In descriptive observation, it means that one observes anything and everything, assuming that he/she knows nothing, however, it can lead to the collection of minutiae that may or may not be relevant to the study. The second type, focused observation, emphasizes observation supported by interviews, in which the participants' insights guide the researcher's decisions about what to observe. In selective observation, the researcher focuses on different types of activities to help delineate the differences in those activities [23].

Here are these three types of the main processes of conducting observations in this study. On the first day of entering each field, the researcher conducted a descriptive observation, selected suitable observation objects, and communicated with them to establish a trust relationship. Focus observation began when the researchers followed the objects in bedside care, to truly observe and experience the characteristics of the empathic labor of hospice nurses, the researchers also conducted further communication with nurses about some meaningful or special phenomena observed in the observation process. Selective observation began later, and special cases such as “How do nurses empathize in grief counseling?” were selected when the obtained data were not sufficiently saturated. The daily observation time of the researcher was consistent with the working times of the objects. Field notes covered details on the field environment and the whole process of nurses' empathy, which included performance and changes in the behavior, communication, and expressions of both nurses and patients.

### Multicenter interview

Hospice nurses who met the inclusion criteria were selected by using purposive sampling, and willing participants from eight cities in southern, central, and northern China were invited to participate in the in-depth, semi-structured, one-time one-on-one interviews [24]. Before the formal interviews, we first conducted a pre-interview with two participants, and finally defined the interview outline as follows: (1) What do you think of empathy in hospice care? (2) How do you empathize with terminal patients? (3) What can affect your “empathic labor” process? (4) Does the experience of empathy with terminal patients have an impact on your work and life, and if so, what are those impacts? (5) What can help you better empathize with terminal patients? The interviews duration range was 30 to 60 min.

### Participants

Those who were willing to participate were required to sign a consent form. All the participants, including hospice nurses from both the observation and interview settings, were recruited if they met the following inclusion criteria: (1) qualified of hospice care and more than one year of hospice care experience; and (2) was aware of the concept of empathy, that is, nurses could briefly and accurately describe the concept of empathy in the clinic using their own words, or could smoothly describe how to use empathy in hospice care. The exclusion criteria were as follows: (1) nurses who were not in the period of rotation of hospice care or had departed this work; and (2) refresher nurses and student nurses. According to data saturation [25], eight hospice nurses were recruited for the field observations, and 26 hospice nurses from 12 institutions in eight cities were recruited for the interviews.

### Data analysis

As we wanted to develop the framework of “empathic labor” based on the theory of empathy and emotional labor, directed content analysis was used as a method for data analysis. Directed content analysis is an approach used in descriptive qualitative studies that validates or expands on an existing theoretical framework or theory and differs from conventional content analysis in its usage of pre-determined broad categories for analysis [26]. The transcribed data were coded independently by the first author [YL W] and another author [ZW Y], they had to read and reread the data many times to become familiar with them before coding, meaning units and coding structure were derived from the interview outline for data reliability and validity [27].

The category of the “empathic labor” process was formed from the theory of empathy, and the sub-categories of affective empathy appeared from the theory of emotional labor. However, some data such as “empathy outcome” cannot use the existing coding scheme. We gave it a new coding and modified coding scheme and then reclassified it to form new categories and sub-categories. Overall, both theme acquisition and qualitative emphasis were determined by research group consensus. When the two separate coders had different topics or multiple themes were difficult to compile into one, they were determined by group consensus. [HL L] inspected the transcripts and themes at each coding stage independently to ensure the integrity and accuracy of the derived themes with interviews.

### Rigor

The rigor of a qualitative study refers to its credibility, transferability, dependability, and conformability, and we used several methods to enhance the rigor in this study [28]. First, intramethod triangulation was employed by using the methodological techniques of field observation and in-depth interview; the research team tried to carry out the observation from different field sites, also interviewed hospice nurses in different cities from southern, central, and northern of China, so as to improve the scientific rigor in the process of data production and analysis and obtain a comprehensive view of Chinese hospice nurses’ empathy characteristics in the context of emotional labor theory. Second, all members of the research reviewed the data set and were involved in the data analysis. Third, verbatim quotations from the informants were provided to improve the transferability and credibility of the study. In addition, collecting qualitative data in one language and presenting the findings in another involves researchers taking translation–related decisions that have a direct impact on the trustworthiness of the research and its report [29]. Therefore, translation procedures in this research can be summarized as follows: two bilingual researchers who were fluent in both Chinese and English translated all original emerged categories independently and compared translation differences until the final English version was reached by agreement between them. Another bilingual researcher was asked to back translate the final English version from English to Chinese. These initial two steps were repeated as necessary to reduce any discrepancies that existed between the original version and the back-translation, and all members of the research need to reach final agreement on the translation.

## Results

### Characteristics of the participants

The characteristics of the participants are reported in Table 1.

**Table 1:** Characteristic of the participants (*n* = 26)

Nurses	Gender	Age	Education	Title	Work experience (years)	Region
N1	Female	43	bachelor’s degree	supervisor nurse	2	Shanghai
N2	Female	36	bachelor’s degree	supervisor nurse	6	Shanghai
N3	Female	39	bachelor’s degree	supervisor nurse	5	Shanghai
N4	Female	41	bachelor’s degree	senior nurse	9	Shanghai
N5	Female	35	master’s degree	supervisor nurse	4	Shanghai
N6	Female	26	bachelor’s degree	senior nurse	3	Changzhou
N7	Female	33	bachelor’s degree	supervisor nurse	8	Changzhou
N8	Female	43	bachelor’s degree	associate professor of nursing	8	Changzhou
N9	Female	40	bachelor’s degree	supervisor nurse	8	Changzhou
N10	Female	51	bachelor’s degree	professor of nursing	8	Lianyungang
N11	Female	35	bachelor’s degree	supervisor nurse	3	Lianyungang
N12	Female	36	bachelor’s degree	supervisor nurse	3	Lianyungang
N13	Female	35	bachelor’s degree	supervisor nurse	1	Lianyungang
N14	Female	39	bachelor’s degree	supervisor nurse	1	Lianyungang
N15	Female	34	bachelor’s degree	supervisor nurse	1	Lianyungang
N16	Female	59	bachelor’s degree	professor of nursing	5	Shijiazhuang
N17	Female	50	bachelor’s degree	associate professor of nursing	4	Shijiazhuang
N18	Female	45	bachelor’s degree	supervisor nurse	3	Shijiazhuang
N19	Female	50	bachelor’s degree	professor of nursing	5	Suzhou
N20	Female	56	associate’s degree (3 years)	supervisor nurse	15	Suzhou
N21	Female	45	bachelor’s degree	professor of nursing	15	Suzhou
N22	Female	41	bachelor’s degree	associate professor of nursing	10	Suzhou
N23	Female	31	master’s degree	supervisor nurse	10	Suzhou
N24	Female	56	associate’s degree (3 years)	senior nurse	7	Beijing
N25	Male	32	bachelor’s degree	supervisor nurse	2	Shenzhen
N26	Female	48	bachelor’s degree	professor of nursing	10	Nanjing

### Experience of “empathic labor” in hospice nurses

**Fig. 1:**
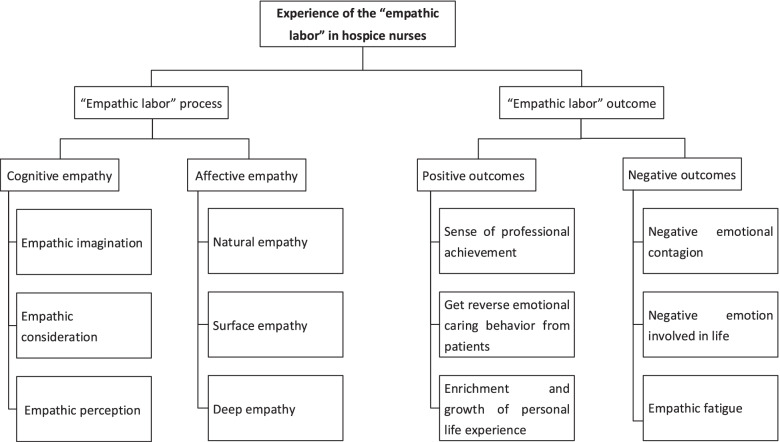
Coding tree

Categories were identified according to the theory of empathy and emotional labor. Each category had two to three sub-categories, which were exemplified by participants' narrative examples (see Fig. 1).

### Category 1: “empathic labor” process

The first category describes the characteristics of Chinese hospice nurses empathizing with terminal patients during the “empathic labor” process. According to the theory of empathy and the research data, “empathic labor” as a main category could be defined as the ‘‘cognitive empathy” and “affective empathy”.

### Sub-category 1: cognitive empathy

The first sub-category, “cognitive empathy”, shows the ability of hospice nurses to perceive and understand terminal patients’ emotions, behaviors, and experiences.

### Empathic imagination: imagine the patients’ role and integrate into it

At the beginning of empathy, hospice nurses should imagine the patients’ role and integrate it since they first visit the patients, which is the basis of establishing a trusted nurse-patient relationship.

“When I empathize with terminal patients, I will first integrate into the role of patients and their families.” (N16)

Under these circumstances, to better convey empathic care to patients, nurses will change their roles according to different empathic objects.

“If you want to enter the patients' heart when empathizing with them, you should constantly change your role and remind yourself that you are not just a medical staff.” (N19)

### Empathic consideration: consider problem from perspective of patients

Most dying patients have different mentalities and behaviors from ordinary people. Many hospice nurses stated that they can empathize with patients by considering the problem from the perspective of the patients and understanding patients’ thoughts and behaviors.

“My biggest professional characteristic is that I am good at putting myself in the patient's position and thinking about many things. You can imagine that if it's our relatives or ourselves lying in the hospital bed, what do we want others to do to me?” (N3)

### Empathic perception: perceive patients’ experiences and discover their needs

Furthermore, some nurses indicated that they could even perceive the pain of patients suffering from illness, just as if they had experienced it. Therefore, they can discover patients’ real needs in time and then will be eager to help patients alleviate their pain.

“When I empathize with patients, I have the same sense as if I had gone through their situation personally, that is, I can perceive the pain of patients in all aspects, including psychology, physiology, and so on.” (N21)

“I can't bear to see the patients and their families in that kind of miserable pain and helplessness, I want to do everything possible to help them.” (N15)

### Sub-category 2: affective empathy

“Affective empathy” is the ability of hospice nurses to emotionally attune to or resonate with the patients’ experiences. According to the theory of emotional labor and the research data, it could be defined as the natural empathy, surface empathy, and deep empathy.

### Natural empathy

Natural empathy means that no emotional conflict exists in the process of empathy between hospice nurses and patients, and the emotional response of nurses is true and natural. When natural empathy occurs, nurses can meet the requirements of professional norms without adjusting their emotional responses.

According to the field data from Lianyungang, one of the notes was recorded as, “Today is the Lantern Festival. However, the patients were unable to reunite with their families because of the COVID-19 prevention and control. To let the patients leave no regrets at the end of their life, nurses cooked dumplings and sent them to the patients’ bedside to for celebrating the festival with the patients. Nurses’ holiday companionship conveys the warmth of ‘home’ to the hearts of patients. The whole process is full of happiness, and the emotional response of nurses is natural and unmodified.”

### Surface empathy

When there is a conflict between nurses and patients in the process of empathy, some nurses’ emotional responses will take the form of surface empathy. Our study found that in this empathic expression, nurses who always face negative emotional events from patients have to passively inhibit their emotional release, making their explicit behavior and emotional response conform to professional norms; however, their internal emotion and sense of value have not changed. This surface empathy of hospice nurses can manifest in the following two situations in this research.

One situation is that when hospice nurses passively accept a bad temper from patients, they will choose to hide their true feelings and try their best to cater to the feelings of the patients, so they will show appropriate explicit emotions that are inconsistent with their inner feelings.

Field notes as follows, “Nurse N15 could keep calm and pretend not to be angry for a moment while she encountered the patient’s bad temper. However, she felt that this patient was unreasonable when she returned to the nurse station, and in fact, she felt very wronged.”

Another situation is that our hospice nurses face a group of dying patients and may face the emotional impact of the patients’ death at any time. This working environment is inconsistent with their original professional belief of "saving the dying and healing the wounded". Therefore, when some nurses face sob stories during the process of empathy with patients, they may pretend to be calm, endure sadness, or even force a smile until the end of the care process.

Field notes as follows, “The patient’s condition worsened again, although the nurse N25 seemed calm to wipe the gushing blood from patient's mouth, I saw his eyes filled with tears and he was trying not to let the tears fall……when he returned to the nurse station, he told me that: ‘Sometimes, if the symptoms of my patients are not well controlled, although I should show calm and insist on completing the nursing work while caring for patients, actually I am very depressed and want to cry with them.’ ”

### Deep empathy

In this study, deep empathy also occurred when there was a conflict between nurses and patients in the process of empathy. The difference is that nurses who can deeply empathize with patients can often actively adjust their inner emotions, understand and help patients from the heart, and respond to negative emotional events with a rational and professional attitude.

“Patients’ bad temper is only temporary, don't be angry about it and argue with them. I can treat patients’ anger more objectively and rationally during the deep empathy process… There must be a reason why the patients get angry, and we should discover their deep needs through empathy with them, rather than just seeing the appearance of the patient losing his temper.”(N23)

Field notes as follows, “Nurse N13 is not afraid of her patient’s ‘cold violence’. On the contrary, she understood that the patient was very young, and the reason why the patient showed long-term bad temper was that the disease disrupts patient’s career and life. Therefore, as long as the nurse N13 has time, she will accompany and communicate with the patient, gradually, she entered the patient’s inner heart.”

Meanwhile, we also found that nurses with deep empathy have the altruistic behavior of spontaneously helping patients to meet their unmet needs which were perceived from cognitive empathy.

Field notes as follows, “The patient was dying, and nurse N20 perceived that what the patient most wanted to see was his son abroad. However, his son was still isolated because of the COVID-19 epidemic. Today, in order to let the patient die without regrets, nurse N3 used many methods to help them meet, including communicating with the epidemic prevention and control department to discuss the isolation time of the patient’s son, accompanying and encouraging the patient, helping them to have a video call, etc.”

### Category 2: “empathic labor” outcomes

According to the participants, they can have different outcome experiences of empathizing with terminal patients, such as inspired from patients’ life experiences during the empathy process, or even depressed from it, and in general, we can summarize those outcomes as positive and negative outcomes.

### Sub-category 1: positive outcomes

Most participants indicated that they can have a deeper understanding of the true meaning of life and death, or have beneficial experiences in their professional and life values during the empathy process.

### Sense of professional achievement

Empathy can help nurses better understand patients’ life experiences and perceive patients’ needs, their altruistic behavior will be motivated and they are willing to provide more comfortable care to satisfy patients’ caring needs.

“With empathy, I found that this patient cared about her hygiene very much and didn't like our oral care liquid and even resisted it. For that reason, I consulted the literature and relevant experts, and finally made flower tea into oral care solution to let her feel more comfortable.”(N11)

Therefore, nurses can improve their nursing quality, and their nursing work can be approved by patients and caregivers. Finally, they can realize their professional value and gain a sense of professional achievement at the end of a nursing process.

“Now I can establish deep feelings with my patients through empathy. They want to tell me anything, and their caregivers also like and trust me.”(N3)

Field notes as follows, “Today, the patient said thank you to the nurse N10 on his deathbed. His family were not in extremely mournful, but calmly accepted the fact that the patient died, and then the nurse N10 told me that ‘It is very meaningful when the patient says thank you to me before he closed his eyes, so I think it is greater to accompany dying patients with empathy, which will bring me a certain sense of achievement.’ ”

### Get reverse emotional caring behavior from patients

Our findings revealed that the empathy path between nurses and patients can be bidirectional, which means that nurses’ empathy can lead to patients’ reverse emotional caring behavior, including considerate words and supportive actions.

“Patients can understand everything I do for them when I establish an empathic bond with them. Meanwhile, they can even comfort me, understand me, help me and tolerate me. In order not to disturb me and reduce my workload, they do what they can do by themselves.” (N7)

In addition, this reverse emotional caring behavior can promote the spread of altruistic culture in society, and produce a self-transcendent function.

“Most families were greatly impressed by the concern we had shown them during the empathy process, many of them want to support us reversely, and some even come to our ward as volunteers to care for more dying people.” (N22)

### Enrichment and growth from personal life experiences

Most nurses indicated that they can benefit and learn from the life experiences of the patients they cared for through empathy, which is conducive to their self-growth and promotes their outlook on life values.

“Now I have become more able to calmly face life and death, more open-minded and indifferent to fame and wealth since I went through too many stories about patients’ death.” (N10)

Some nurses expressed that they became more able to cherish their life and family and willing to spend time with and care for their family members.

“I cherish what I have now, care more about my family, and respect the meaning of my life.” (N12)

### Sub-category 2: negative outcomes

However, our findings found that when nurses consider problems from the perspective of the patients’ roles during the empathy process, they will also fall into patients’ negative emotions, even resulting in certain emotional trauma.

### Negative emotional contagion

Some participants said that they were easily infected by patients’ bad emotions and came into bad moods such as sadness and loss. As time passed, their emotions became vulnerable and fragile for suffering from those sad events for a long time.

Field notes as follows, “When the nurse N11 saw this child, she said she suddenly thought of her own child, and at that time, she couldn't control her tears when she saw this little child suffering from the illness.”

“I was immersed in this lost mood for several days during the period when my patient died. I even felt more depressed and thought of him when I walked to the door of his ward.” (N25)

More seriously, some nurses expressed that it is difficult for them to find ways to pour out their bad emotions, and most of them chose to endure these bad experiences, which may even cause them to have psychological diseases such as depression.

“My family and friends don’t want to listen to my experience of hospice work, so I won’t tell them so as not to upset them.” (N12)

“I really can’t find a way to relieve my mood, and now I also feel a little depressed.” (N20)

### Negative emotion involved in life

Meanwhile, when nurses cannot adjust those negative emotions in time, they may unconsciously bring those bad emotions into their lives, which can destroy the harmony of their family relations and reduce the quality of their personal lives.

Field notes as follows, “Unfortunately, the patient died in pain two weeks ago, but today, nurse N13 still can’t get out of the empathy state with him. She said she has seen several psychologists, but it was useless.”

“If those bad emotions can’t be released, I will still be in a bad mood when I get home. I even can’t help losing my temper with my family sometimes, so my family relations will also be affected.” (N9)

### Empathic fatigue

Nurses need to spend much time accompanying and listening to patients during the empathy process; however, their long-term emotional investment will easily cause them to lose sensitivity and motivation to empathize with others. In the long run, it is easy to cause nurses to feel empathic fatigue and even empathic exhaustion.

“The state of patients willing to give their life history to you without hesitation cannot be achieved in a day or two. For example, you may say 10 words before the patient is willing to answer you at the beginning of nursing work…I will control myself not to have the self-emotions opposite to my patient and find ways to go into the patient’s heart. Therefore, when I follow a patient from beginning to end, I really feel exhausted and I have no patience to stick to it.”(N15)

## Discussion

Empathy is an institutional norm in medicine, and the empathic relationships between nurses and patients are a process that occurs most often over time in the field of hospice care [30]. This study described the process of empathy in hospice nurses by combining emotional labor and empathy theory and also analyzed the definition and features of nurses’ empathy through two components of cognition and affection.

Cognitive empathy is the ability to think into or mentally reconstruct the other's experiences, mainly by using one’s imagination [7]. From this definition of cognitive empathy, empathic imagination is an important ability that requires nurses to imagine and share similar thoughts and feelings with their patients [31]. In this study, we developed this definition into three dimensions to be suitable for a hospice care environment. First, we considered empathic imagination to include hospice nurses imagining the patients’ role and integrating into it. Second, with empathic consideration, nurses need to consider about caring problems from the perspective of the patients. Finally, empathic perception emphasizes the requirement of nurses to perceive patients’ experiences and discover their unmet needs in daily work. This is because the purpose of hospice care is to help terminal patients go through their lives without regrets. Therefore, when hospice nurses empathize with patients, in addition to having the ability to imagine the patients’ role and consider from patients’ perspective, nurses’ ability to accurately perceive the patients’ end-of-life care needs is even more important. However, based on previous studies, most focused on the imagination dimension of cognitive empathy, therefore, it is not enough to imagine the experience of dying patients, and hospice nurses should be cultivated to raise their occupational sensibility of patient-centered consideration and their perception of patients’ unmet needs through empathy. Strategies include taking a target’s perspective, reading facial expressions, developing related curricula training programs, and accessing memories of relevant previous situations [32, 33].

The field of medical education has focused on the cognitive aspects of empathy, neglecting the affective components [34]. Empathy is also a clinical skill that can be used as a strategy to effectively create patients’ feelings of satisfaction and comfort; thus, it is a form of medical work that should be understood as emotional labor [11]. Therefore, this study focused on exploring the characteristics of nurses’ affective empathy under a hospice caring environment, considered it emotional labor, and found that nurses’ empathy is also an emotional labor process, including dimensions of natural empathy, surface empathy, and deep empathy. This may be because the exploration of training for palliative care specialist nurses in mainland China is insufficient [35], and there is no standardized hospice care curriculum education in various medical colleges and universities. When the government launched a pilot project of hospice care in 2017, most nurses engaged in hospice were transferred from other departments and carried out hospice care after short training. This may lead to the uneven empathy ability among nurses, and therefore, display these three different dimensions of performance in the empathic labor process.

Nurses with surface empathic labor can easily suppress their true feelings and force themselves to smile or resist anger in front of patients. However, nurses who have the ability of deep empathy with patients can self-adjust their inner emotional state, analyze the root causes of patients’ bad emotions or behaviors, subtly perceive patients' unmet caring needs, spontaneously provide humanized care for patients, help patients achieve their last wishes, and improve the quality of terminal care for dying patients. Different from those in the working environments of other clinical departments, hospice nurses always need to devote much emotional labor because they often face more complex psychosocial events, such as patients' severe pain, death, and bereavement [36]. Therefore, we should not only pay attention to nurses’ affective components of empathy but also cultivate nurses' affective empathy to reach the level of deep empathy. Senior staff nurses can be set as good examples and encouraged to share their empathy cases to strengthen the humanistic quality and self-emotional regulation ability of young nurses and improve their ability to deal with various situations encountered in hospice care with positive beliefs and flexible methods. Thus, nurses’ deep empathic labor can have a more positive effect, including improving patients' nursing satisfaction, and is conducive to nurses' physical and mental health.

In addition, we also summarized the outcomes of nurses’ empathy to provide a basis for their empathy empowerment in the future. Our research found that the empathy of hospice nurses has important positive significance. For the nurses themselves. First, empathy can help them improve their nursing quality of hospice work by being more sensitive to patients’ pain and needs, stimulating their active altruistic behavior, and providing more comfortable care for dying patients. Under these circumstances, they will obtain affirmation and support from patients or their families, and will gain the sense of professional achievement. Second, empathy can enrich nurses’ personal life experiences and help them change to a positive attitude toward life. This is because nurses can learn about life, cherish life, and reshape their view of life by listening to and observing the life experience of patients during the daily empathy process with patients. For patients, empathy always involves reciprocal sharing, which can help patients understand nurses’ nursing work and provide emotional feedback. This may be because empathy itself is altruistic, nurses’ empathic concern can touch patients and their family members and make them willing to pay back in the form of volunteers spontaneously. Therefore, the positive significance of empathy can be incorporated into the quality management of hospice care to comprehensively evaluate nurses’ hospice work.

Despite these salutary gains as the result of successful empathy, the negative impact of empathy cannot be ignored. Contrary to the traditional professional belief of “healing the wounded and rescuing the dying”, the professional belief of hospice nurses is often challenged because they have long been exposed to shock events such as patient death. Therefore, it is common for them to be accompanied by sadness, depression, and other emotions. When these negative emotions are difficult to remove, nurses may lose themselves in emotional trauma which will endanger their physical and mental health. If this persists, it will even have an impact on nurses’ families or their nursing quality. Furthermore, previous research points out that hospice nurses are more prone to empathic fatigue than nurses in other departments [37]. This may easily to make nurses lack empathic power and become indifferent to their patients and work, or even make nurses have a turnover intention [17], thus impacting the quality and safety of their hospice work and the stability of the hospice nursing team. Therefore, we should design and implement scientific empowerment strategies to help nurses intervene in the negative experience of empathy by listening to and caring for nurses, guiding nurses to learn emotion control and decompression skills, strengthening the construction of nurses' positive psychological resources, etc.

This study has some limitations. As a qualitative study, we only explored the experience of empathy in hospice nurses, and the relationship between nurses' empathy ability and different influencing outcomes could not be explained in this study. In addition, it was specific to a small number of participants within a particular setting, and the sensitivity of some questions during the interview may interfere with nurses' expansion of the conversation; therefore, we have not found the phenomenon of hospice nurses’ turnover intention because of their empathic fatigue, as mentioned in other studies.

### Implications for practice and research

At present, hospice care in China is still in the initial stage of development. Nursing colleagues pay more attention to the construction of the hospice care model, and few policies bring empathy into the core competence cultivation system of hospice nurses. This study creatively discussed the importance of nurses' empathy from the perspective of emotional labor theory, aimed to call on nursing managers to pay more attention to the cultivation of nurses' deep empathy, and explored the strategies of how nurses can empathize effectively.

## Conclusion

In this study, empathy is an important clinical skill that can effectively create patients’ feelings of satisfaction and comfort, and nurses’ empathy process should be understood as emotional labor. We should pay attention to the ability level of the empathic labor of hospice nurses, give more sufficient active learning channels and training chances to strengthen nurses' deep empathy with terminal patients, and explore more sufficient active empowerment strategies to alleviate the negative impact of empathy on nurses.

## Data Availability

The datasets used and/or analyzed in this study are available from the corresponding author upon reasonable request.
